# High variability orthographic training: Learning words in a logographic script through training with multiple typefaces

**DOI:** 10.3758/s13423-025-02646-0

**Published:** 2025-03-17

**Authors:** Eric Pelzl

**Affiliations:** 1 The Hong Kong Polytechnic University, Hung Hom, Kowloon, Hong Kong; 2 The Pennsylvania State University, University Park, PA USA

**Keywords:** Vocabulary learning, High variability training, Desirable difficulty, Orthography

## Abstract

We tested whether naturally occurring visual variability—specifically, typefaces—would help people generalize word learning to typefaces they had never seen before. In Chinese, thousands of unique written characters must be learned item by item, and differentiated from similar-looking characters. Participants (*n* = 190) with no previous Chinese experience learned 24 Chinese characters in one of two training groups: the Single-Typeface group trained using only one of three Chinese typefaces; the Variable-Typeface group trained using all three. Everyone completed two training and testing phases. During Definition Training, they saw each character six times and learned to associate it with an English definition (水–water). After training, participants were tested on their accuracy in providing definitions for the characters. During Form Training, participants chose the characters they had previously learned from a display that included a trained character and a visually similar distractor (水 vs 永). After training, they were tested on their speed/accuracy in choosing the learned characters. At testing in both phases, half of the words were presented in a familiar typeface; half in a novel typeface. Results showed significant interactions between training and testing conditions in both phases, with a significant effect of training in the Form Testing phase: Single-Typeface training resulted in faster responses for familiar typefaces, but much slower responses for novel typefaces; in comparison, Variable-Typeface training resulted in better generalization to novel typefaces. These results suggest that typeface variability can influence how effectively people generalize knowledge during the initial stages of learning a logographic script.

**Supplementary Information:**

The online version contains supplementary material available at 10.3758/s13423-025-02646-0.

Across many domains of human learning, training outcomes are often improved by the inclusion of variability in training conditions (for a review, see Raviv et al., 2022; for a caveat, see Bowman & Zeithamova, 2023). Whereas training with little or no variability tends to produce faster initial learning progress, the learning is typically narrow and may not generalize well beyond the specific conditions encountered during training. In contrast, when training incorporates more variability, initial progress may be slower, but the knowledge gained is more flexible and generalizes more easily to novel conditions not encountered during training.

In studies of spoken language learning, so-called High Variability Phonetic Training (HVPT) has inspired decades of research (for reviews, see Brekelmans et al., 2022; Thomson, 2018; Zhang et al., 2021). Seminal studies by Logan and colleagues (Lively et al., 1993; Logan et al., 1991) suggested that using multiple speakers and words during training could help Japanese learners of English generalize their learning of the English /l/ and /r/ to novel words and novel talkers (for a well-powered failure to replicate those seminal results, see Brekelmans et al., 2022). Based on those findings, many training studies have incorporated variability, either testing or assuming its benefits for various aspects of phonetic learning. A related line of research has also suggested that some types of phonetic variability can improve retention of newly learned second language vocabulary (e.g., Barcroft & Sommers, 2005; Sommers & Barcroft, 2011).

The present study pursues a similar line of investigation, but in the visual realm, testing how natural variability in the forms of written symbols impacts written language learning. Perhaps, by analogy with HVPT, we might call it High Variability *Orthographic* Training (HVOT). This is somewhat of a misnomer for the present study, however, which does not directly contrast *high* versus *low* levels of variability, but *variability* versus *consistency* in training conditions (in fact, a common design for HVPT studies as well). Specifically, we investigated whether the impacts of training variability extend to typefaces (fonts). Although fluent readers rarely notice the variation that occurs between typefaces, it can be substantial. For example, the basic shape of the lowercase “G” as it appears in Times New Roman (< g >) versus Helvetica (<  >) is quite different, not to mention is quite different, not to mention) is quite different, not to mention the thickness and ornamentation of strokes. Such typeface variability might initially be surprising or confusing for novice readers. Could it also be leveraged as a tool for their learning?

To investigate this question, we examined how training with a variety of typefaces impacted the learning of Chinese characters by adults with no previous Chinese language experience. As will be seen below, we found evidence for small but clear impacts of training with variable typefaces. This study serves as a proof-of-concept that suggests a new space for testing theoretical claims about the role of variability on naturalistic human learning. Additionally, it could have practical applications for the teaching and learning of writing systems, especially of logographic scripts like Chinese.

## Variability in written symbols

Variability in written systems is realized at multiple levels. Writing systems themselves form one type of category that can vary (English vs Chinese; alphabetic vs logographic). Within each system are the written symbols themselves (letters, characters), each distinctive from the others (d, b, p, q; 大, 天, 关, 美). Layered on these symbols are differences in visual style, such as occur in handwriting or across typefaces. Importantly, this stylistic variability is a type of within-category variation for the written symbols or writing systems, but is also a type of between-category variation at the level of style. It is the potential utility of this stylistic visual variability which is of interest in the present study.

Typeface variability can be considered a type of heterogeneous variability, where instances of the same visual category vary across training (see Raviv et al., 2022, for four types of training variability). Heterogeneity itself is a matter of degree. Typefaces can be more or less similar to one another such that the degree of heterogeneity could be smaller or larger depending on the specific typefaces used. In other words, along with heterogeneity among typefaces, their degree of similarity or difference might also matter.

Research of variability in visual category learning has a rich history, often focused on how more or less variability effects binary category learning, and how outcomes align with specific theoretical models (e.g., prototype vs exemplar; for a review, see Minda et al., 2024). Although influential early studies were originally interpreted as supporting high variability over low variability (e.g., Posner & Keele, 1968), subsequent research has instead indicated that, for binary category learning, less (but not no) variability is superior to more (e.g., Bowman & Zeithamova, 2023; Hintzman, 1986; Hu & Nosofsky, 2024), and that the similarity of training exemplars to the category prototype is highly important. We will return to these issues later in the discussion.

Although visual variability is a common domain of study, specific consideration of typeface variability has been only an occasional feature of previous research. In the case of alphabets, a key learning outcome is the formation of associations between a given letter and the sound(s) it represents, so-called *grapheme-to-phoneme correspondences* (e.g., Castles et al., 2018). For example, readers of English need to know that the letter < a > often represents the vowel /æ/ (as in < cat >). Research on variability in this area has been motivated in part by the apparent connection between early handwriting practice and later literacy outcomes (James, 2017; Seyll et al., 2020). While it is often assumed the benefits of handwriting are causally linked to the use of motor skills, Li and James (2016) considered whether these apparent benefits might instead be due to the variability of the exemplars produced when children write by hand. They trained American kindergarteners to learn four Greek symbols in a variety of conditions including hand-writing practice or exposure to variable typefaces (without hand-writing practice) and found that variability in typefaces produced similar gains as hand-writing practice. Along similar lines—but with quite different outcomes—Wiley and Rapp (2021) trained adults in recognition of Arabic letters using typing, visual only training (with variable typefaces), and handwriting training. They found that handwriting was both more efficient (required fewer repetitions) and produced better learning outcomes than either typing or visual only training. Neither of these studies included a single typeface condition, so results did not speak directly to the impact of variability per se, but only variability relative to handwriting.

In sum, while typeface variability has occasionally appeared in the context of research on alphabetic learning, these studies have not specifically aimed to test whether more or less variability leads to different learning outcomes. Perhaps this is due to the relatively limited set of written symbols that comprise most alphabets, making the task of learning the symbols themselves seem fairly straightforward. For Chinese, of course, this is not the case.

## Chinese characters

Chinese characters are variously labeled logographic (Sampson, 2015), or morphosyllabic (DeFrancis, 1989). A single character maps to a single syllable, and because most Chinese morphemes are monosyllabic, a single character also typically maps to a single morpheme. Many morphemes can stand on their own as words; in such cases, to learn a character is to learn a word. As an illustration, the character < 水 > represents the morpheme/word that is pronounced as /ʂuei3/ and means ‘water’. Whereas readers of alphabetic systems need to form grapheme-to-phoneme correspondences, readers of morphosyllabic systems need to form grapheme-to-*morpheme* correspondences. Because morphemes are both phonetic and semantic, fully learning a Chinese character means knowing a three-way relationship between symbol, sound, and meaning (in the current study, sound relations will not be addressed).

Chinese characters are renowned for their visual complexity. Traditionally, the structure of a character is described according to its *strokes*—that is, a line or dot that would be made with a single fluid movement of a brush/pen. While the simplest characters can consist of a single stroke (e.g., 一 ‘one’), many have ten or more strokes (e.g., 鼠 ‘mouse’). Characters can often be decomposed into components and sub-components that re-occur across the written system in more or less systematic ways (Myers, 2019). Individual characters are also often used as semantic or phonetic components in other characters. For instance, the character 女 (/ny3/ ‘woman’) and the character 马 (/ma3/ ‘horse’) are combined to indicate the meaning and sound of the character/morpheme 妈 (/ma1/ ‘mom’). Thus, despite potential universal principles that may apply to reading in any language (Verhoeven & Perfetti, 2022), there are also distinctive characteristics of logographic writing that present a novel learning task for readers who have previously only learned alphabetic writing systems.

In addition to being visually complex, characters are also numerous. To be literate in Chinese, one must learn thousands of characters. A rough estimate is that knowledge of around 3,000 characters is necessary for basic literacy (Kubler, 2006). Educated Chinese readers certainly know more than that. One study has suggested the average college graduate might know around 5,150 characters (Hue, 2003). The SUBTLEX-CH corpus (Cai & Brysbaert, 2010) compiled from 33.5 million words used in Chinese subtitles includes 5,936 unique characters. So, whether it is 3,000 or 6,000 characters, suffice to say, learning to read Chinese requires a sustained commitment to learning new characters.

Given the number and complexity of Chinese characters, training that might speed up or improve the quality of learning is desirable, and even small effects have the potential to translate to substantial benefits over time.

## Chinese typefaces

Just like alphabetic systems, Chinese has developed a variety of typefaces that are commonly used in written materials and, despite sometimes quite dramatic visual differences among typefaces, do not typically pose difficulties for fluent Chinese readers.

Chinese typefaces vary for numerous reasons, whether it be stylistic (e.g., to imitate calligraphy), or pragmatic (e.g., to fit within a specified grid of pixels). The same character may be realized with strokes that differ in thickness, angle, curvature, length, relative alignment with other strokes, distance from other strokes, and ornamentation at stroke ends. Importantly, some of these elements of variability can also differ in whether or not they are relevant for distinguishing one character from another. Whereas spacing is not relevant between the horizontal stroke and the vertical strokes in the middle and right side of 瓜 (*guā* ‘melon’)—and indeed varies among typefaces (e.g., 瓜vs 瓜)—a space between the right and left sides of 入 (*rù* ‘enter’) would indicate a different character 八 (*bā* ‘eight’) regardless of typeface (入八;入八).

One reason variability in typefaces might be beneficial for learners is that it could draw their attention to these differences. In this way, it might help them to form mental representations of the characters that could flexibly adjust for recognition of the same characters presented in new forms.

## Present study

Utilizing the variability inherent in Chinese typefaces, the present study asks how the use of a single typeface or multiple typefaces during training impacts people’s learning of Chinese characters. We compare the results of participants who learned a set of 24 Chinese characters using a single typeface during training with those of participants who learned the same 24 characters presented in multiple typefaces. We seek to answer two basic questions related to people’s ability to generalize learning to novel instances of characters. First, compared with training with a single typeface, does training with variable typefaces result in better generalization of form-to-meaning associations for words presented in a novel typeface? Second, does it result in faster recognition of previously studied characters presented in novel typefaces?

## Methods

### Participants

Participants were 190 undergraduates at a large research university in the United States (130 women, 59 men, one nonbinary; age *m* = 19.1, *sd* = 1.1). Based on power simulations conducted using *simr* (Green & MacLeod, 2016), this sample size has greater than 90% power to detect smaller main effects and training-by-testing interactions than were found for definition testing outcomes in a pilot study. These participants represent a reasonable sample of the population that might choose to study Chinese in many university classrooms.

During data collection, data quality was monitored and participants were removed prior to further analysis if they met any of the following exclusion criteria: (1) They made a partial attempt before fully completing the experiment [three exclusions]. (2) They provided only one or fewer correct responses across all six blocks of definition training [four exclusions]. (3) They had unrealistically fast average response times (< 500 ms) during Form Training or Form Testing [10 exclusions; average mean RT after exclusions was 1,553 ms]. (4) Their total time to study completion was greater than 90 min [10 exclusions; average completion time after exclusions was 38 min]. (5) Despite confirming on the pre-screening survey that they did *not* know a language with a non-roman orthography, on the post-experiment survey they claimed knowledge of an L1 or L2 with a non-roman orthography [18 exclusions]. (6) They acknowledged previous knowledge of Chinese words or demonstrated it by providing correct definitions for more than one word in the first block of definition training [seven exclusions]. Data collection was stopped when 192 total useable data sets were collected. Three additional participants were excluded due to completing the study after the target sample size was met. After data collection was completed, two further participants were excluded due to problems that were missed during data collection (repeated attempts, failure to complete the final survey). In total, out of 245 people who completed the study online, 55 were excluded (22%).

### Stimuli

**Fig. 1:**
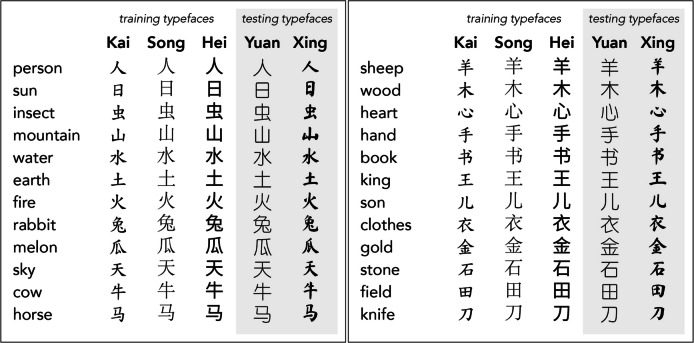
English definitions along with the 24 target Chinese characters displayed in each of the three training and two testing typefaces

Stimuli consisted of 24 common Chinese characters in five different typefaces (Fig. 1). Each character was paired with an English translation that was a concrete noun. Characters were relatively simple, comprising two to eight strokes. For training, three commonly used typeface styles were chosen (Palmer, 2020).^1^*Kǎitǐ* (from now on “Kai”) is based on a calligraphic style that clearly delineates each stroke of a character (i.e., it is not ‘cursive’). *Sòngtǐ* (“Song”) is based on the woodcut style that was the most common form of printing in China until the nineteenth century. *Hēitǐ* (“Hei”) is a modern style developed for digital interfaces. For the novel typefaces, we used *Yuántǐ* (“Yuan”), which is similar to the Hei style, but has a more rounded appearance. We also chose a light (thin) weight to further differentiate it from the Hei typeface. The other novel typeface *Xíngkǎitǐ* (“Xing”) is based on a more flowing calligraphic style with stronger variation between thin and thick lines in imitation of brush strokes, but still clear delineation between individual strokes (again, not cursive).

**Fig. 2:**
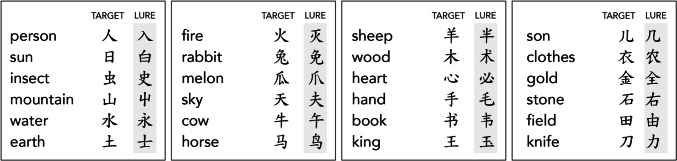
English definitions along with the 24 target Chinese characters and their lures used in Form Training and Testing

In addition to the 24 target characters, another 24 characters were selected to serve as ‘lures’ in Form Training and Testing. Lures were chosen for visual similarity to the target characters (Fig. 2; for the full list of all stimuli in all typefaces, see the Supplementary Materials Appendix A). Lures typically had one or two strokes that differed from the target. Differences included additional strokes, fewer strokes, or variation in the shape or configuration of strokes. With the exception of 屮 (the lure for 山 ‘mountain’), all included characters are frequent in modern Chinese writing. This assessment was based on the intuitions of two experienced L2 Chinese learners. Subsequent referral to the SUBTLEX-CH-CHR corpus (Cai & Brysbaert, 2010) confirmed that all characters had log frequencies above 2.7 [mean = 4.04]. Inspection of the targets and lures will show that some visual differences are easier to detect than others. This reflects the natural variation in differences among characters; no attempt was made to control variation at a more granular level in the present study.

### Procedures

**Fig. 3:**
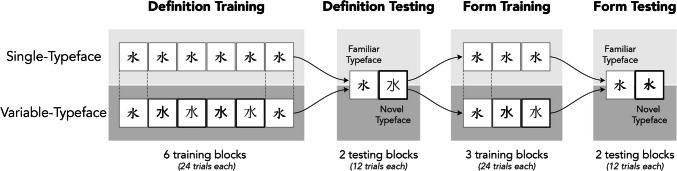
Progression of training and testing phases. The use of typefaces for Single-Typeface and Variable-Typeface groups differed during training phases, but was the same for both groups during testing phases

The study had two distinct training and testing phases (Fig. 3). First participants were trained and tested on word meanings (Definition Training and Testing), then they were trained and tested on their ability to differentiate the previously learned characters from similar looking characters (Form Training and Testing). Participants were randomly and evenly assigned to one of six subgroups (three for Single Typeface training, three for Variable; for further details, see the Supplementary Materials). All procedures were completed online using the Labvanced platform (Finger et al., 2017), with a post-study survey completed in Google Forms.

### Definition training

**Fig. 4:**
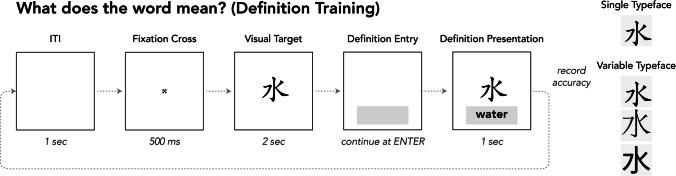
Trial structure and parameters for Definition Training. The structure of Definition Testing was the same except that the character’s definition was not presented (no feedback)

Participants completed six blocks of Definition Training, during which they learned to associate English definitions with Chinese characters. The decision to block typeface presentation, rather than intermix it, was based on the results of Perrachione et al. (2011), which suggested blocked presentation is more beneficial for most learners. Trial parameters for Definition Training are depicted in Fig. 4. Each trial began with a 1-s blank screen followed by a 500-ms fixation cross. A character was displayed for 2 s, and then disappeared, at which point participants were prompted to provide an English definition. In the first block, these responses were guesses and expected to be incorrect. After the participant pressed enter, they saw the character displayed again for 1 s, accompanied by its English definition. For the Single-Typeface group, all words across training blocks were displayed in only one of the three training typefaces. For the Variable-Typeface group, all three typefaces were used, changing in each block, and organized so that the initial and final block used the same typeface as in the corresponding list from Single-Typeface training (see Fig. 3). The motivation for this order was to control for memory benefits that might accrue to participants for the first or most recent typeface.

### Definition testing

After Definition Training, participants immediately completed two blocks of Definition Testing. Trial parameters were the same as those used during training, except that feedback was no longer provided after participants entered definitions. The test phase was the same for both groups. In the first test block, 12 of the trained words were displayed in the Familiar Typeface that had been used in all six training blocks for the Single-Typeface group and two of the training blocks for the Variable-Typeface group. In the second testing block, the other 12 trained words were presented in a Novel Typeface that neither group had previously encountered.

The order of Familiar and Novel Typeface testing blocks was fixed so that the Familiar Typeface was always first, followed by the Novel Typeface. The 24 target characters were randomly assigned to two lists of 12 items. For each participant, one set of 12 characters was displayed in the Familiar Typeface and the other in the Novel Typeface. The assignment of each set of 12 to the Familiar or Novel Typeface was counterbalanced across participants (i.e., the 12 characters that appeared in the Familiar Typeface in List A appeared in the Novel Typeface in List B). Which of the two novel typefaces (Yuan or Xing) was used was also counterbalanced so that if Yuan was used during Definition Testing, Xing was used during Form Testing, and vice versa.

### Form training

**Fig. 5:**
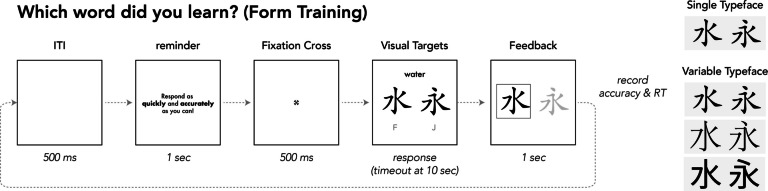
Trial structure and parameters for Form Training. The structure of Form Testing was the same, except that there was no feedback

After Definition Testing, participants proceeded to the Form Training phase. Trial parameters for Form Training are depicted in Fig. 5. On each trial during Form Training, participants were shown an English definition along with two Chinese characters. One of the characters was the *target*—a word that corresponded to the English definition and which they had previously encountered during Definition Training and Testing. The other character was a *lure*—a new character that resembled the target character, but had at least one distinct difference in its written form (e.g., *water:* 水 vs 永). For each trial, the participant had to select the word they had previously learned (the target) by pressing the key that corresponded to the right (“F”) or left (“J”) location on the display. After they made their selection, they were given feedback indicating which of the two words was the target. The location (left/right) of target and lure was random across trials.

Participants completed three blocks of Form Training. Each block included all 24 trained target words and their accompanying lures. For the Single-Typeface group, all Chinese characters were presented in the same typeface as had been used during Definition Training; for the Variable-Typeface group, the three typefaces used during Definition Training were used, with a different typeface in each block. The first block always presented the typeface that would be used as the familiar typeface during testing.

### Form testing

Form Testing followed immediately after Form Training. The procedure was the same as during training except that feedback was no longer provided. As during Definition Testing, words were presented in two blocks of 12. In the first block, the Familiar Typeface was used, in the second block a Novel Typeface (different from that used during Definition Testing) was used. Two lists were created to balance which words appeared in the Novel or Familiar Typeface at test (in this case, six of the words that appeared in the Familiar Typeface during Definition Testing and six of the words that appeared in the Novel Typeface were now presented in the Familiar Typeface in List A, and in the Novel Typeface in List B). Testing was the same for both the Single-Typeface and Variable-Typeface groups.

## Data processing and analysis

### Data processing

#### Data processing: Definition training and testing

Definition Training and Testing data were processed in R (Version 4.0.3; R Core Team, 2022). Definition accuracy was scored automatically using strict matching criteria. Only exact matches were scored ‘correct’ (1). Misspellings and typos (e.g., ‘earht’ for ‘earth’) were marked ‘incorrect’ (0), as were near synonyms (‘clothing’ was not accepted for ‘clothes’). Manual inspection indicated that 47 out of 4,560 test responses (~ 1%) could be considered typos or near synonyms; these responses were quite evenly distributed across participants, training groups, and testing typefaces (Single: Familiar = 11; Variable: Familiar = 13; Single: Novel = 9; Variable: Novel = 14). Notably, some items attracted more typos than others (e.g., ‘mountain’ was misspelled six times, likely because it was longer than most other definitions). An alternative analysis was run counting all 47 items as ‘correct’; results did not differ substantively from those reported below (see supplementary materials for details).

#### Data processing: Form training and testing

Form Training and Testing data were processed in R (Version 4.0.3; R Core Team, 2022). The accuracy of responses was scored ‘correct’ (1) or incorrect (0). Only trials with correct responses were retained for RT analyses (88.1% of trials overall). To better fit the normality assumption, RT data were trimmed of extreme values (RTs 2.5 standard deviations outside of each participant’s mean in each typeface) and log transformed. Trimming resulted in a loss of an additional 1.7% of trials. No additional exclusions were applied. Overall, 3,941 out of 4,560 trials (86.4%) were retained for further RT analysis.

#### Data analysis

Statistical analyses focus on accuracy results in Definition Testing and RT results in Form Testing. Based on a similar task used during piloting, participants were expected to be quite accurate in the Form Testing task, thus accuracy was not considered a critical outcome. In the interest of space, below I only report descriptive statistics for results of Form Testing accuracy (for full statistical analyses of Form Testing accuracy data, as well as additional figures for all analyses, see the supplementary materials).

All analyses were conducted in R (Version 4.0.3; R Core Team, 2022). Accuracy data for Definition was modeled using generalized linear mixed-effects regression, and RT data for Form Testing was modelled using linear mixed-effects regression. All models were fit with the *lme4* (Version 1.1.21, using the BOBYQA optimizer; Bates et al., 2015), and *afex* (Singmann et al., 2022) packages. For Definition Testing data, the dependent variable was Accuracy (1, 0). Fixed effects were sum coded (1, − 1) and included the factors Training Group (Single, Variable), and Typeface Condition (Familiar, Novel). Random intercepts and slopes were included for the effects of participants and items. The fully specified maximal random effects model was fit first (Barr et al., 2013) using the “mixed()” function from *afex*. If a model failed to converge or generated singular fit warnings, it was refit using a zero-correlation parameter, and the random components that generated the smallest variances were dropped. The maximal model that converged with no singular fit warnings was retained as the final model, but was also manually inspected for differences in outcomes compared to the fully specified model. Where differences occurred, this is noted below.

The final model for Definition Testing accuracy included by-subject intercepts and slopes for the effect of typeface condition, and by-item intercepts: mixed/*glmer model formula*: accuracy ~ 1 + training group × testing typeface + (1 + typeface condition | participant) + (1 | item).

For RT data from Form Testing, the dependent variable was log RT. Fixed effects were sum coded (1, − 1) and included the factors Training Group (Single, Variable), and Typeface Condition (Familiar, Novel). Random intercepts and slopes were included for the effects of participants and items. The same model selection procedures were used as outlined above. The final model for Form Testing RTs included by-subject and by-item intercepts and slopes for the effect of typeface condition: *mixed/lmer model formula*: log(RT) ~ 1 + training group × testing typeface + (1 + typeface condition | participant) + (1 + typeface condition | item).

Significance tests of main effects and interactions were obtained from the models using the “anova()” function. Planned comparisons of the critical effects of training group and testing typeface were conducted using the *emmeans* package (Lenth, 2022) with Holm corrections applied to adjust for multiple comparisons.

## Results

### Descriptive results: Definition training and testing

**Fig. 6:**
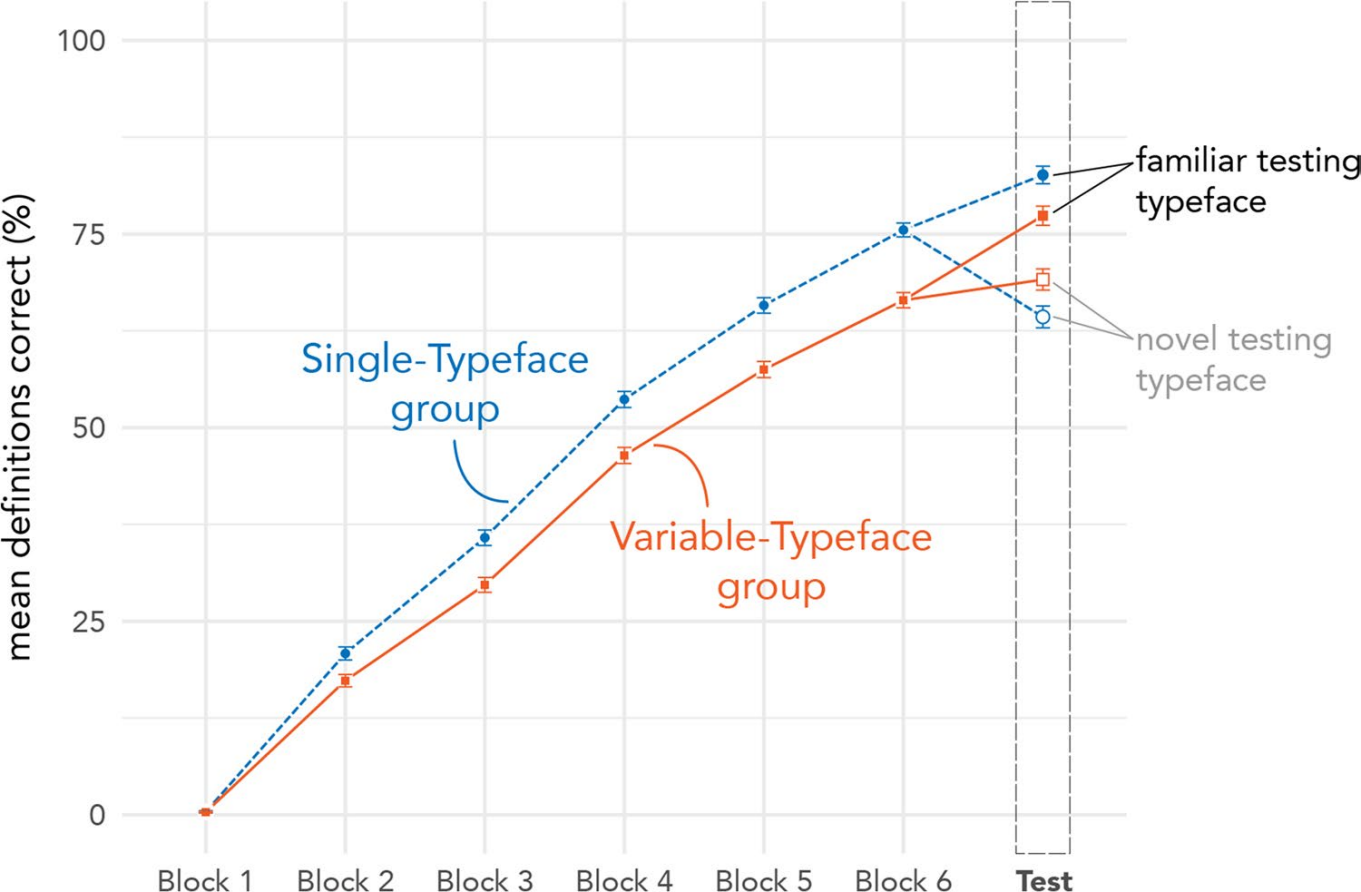
Raw accuracy of definitions across blocks of Definition Training and the Test phase. Error bars depict standard error. (For more detailed figures showing individual participant variability, see the Supplementary Materials)

Raw accuracy results across blocks of Definition Training and Testing are visualized in Fig. 6. Both training types produced steady increases in accuracy across blocks, with Single-Typeface training leading to faster progress overall compared to Variable-Typeface training. At Testing, the Single-Typeface group showed continued improvement for the Familiar Typeface, but a drop in accuracy for the Novel Typeface. In contrast, the Variable-Typeface group showed improvement for both Novel and Familiar Typefaces. Table 1 lists accuracy at test, broken down by training group and typeface condition. An accuracy difference of 8.3% equates to roughly one word (one of 12 words). In other words, the effects observed in Definition Testing suggest differences of one word or less in learning outcomes between training groups and typeface conditions.

**Table 1:** Definition testing accuracy

Training group	Condition	Mean (%)	(*SD*)
Single-Typeface	Familiar	82.6	(37.9)
(*n* = 95)	Novel	64.3	(47.9)
	*Overall*	*73.5*	*(44.2)*
Variable-Typeface	Familiar	77.4	(41.9)
(*n* = 95)	Novel	69.1	(46.2)
	*Overall*	*73.2*	*(44.3)*

### Inferential statistical results: Definition testing

**Fig. 7:**
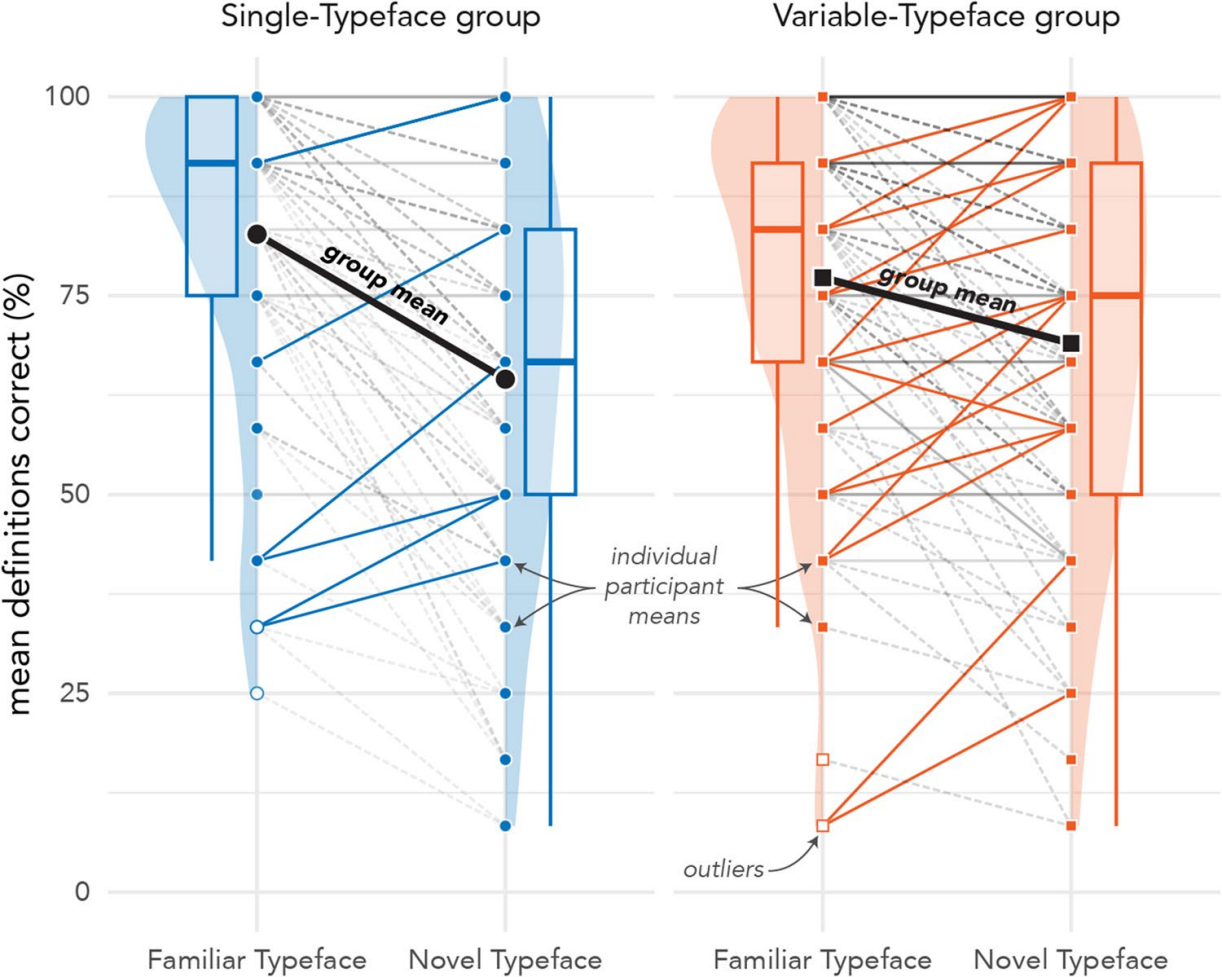
Accuracy by testing typeface for Definition Testing. Single-Typeface group results are pictured on the left, Variable-Typeface on the right. Large black dots indicate group means; smaller dots indicate individual participant means. Lines connect scores for participants/groups in the two testing conditions (Familiar vs Novel Typeface). Colored lines indicate increases in score; dashed lines indicate decreases in score. The distribution of scores is shown in the shaded area for each testing condition, along with boxplots that capture the median score (thick center line) and interquartile range of scores

Mixed-model analysis of variance (ANOVA) results indicated no significant main effect of training group, χ^2^_(1)_ = 0.09, *p* < 0.769, a significant main effect of testing typeface condition, χ^2^_(1)_ = 85.65, *p* < 0.001, and a significant interaction between training group and typeface condition, χ^2^_(1)_ = 17.57, *p* < 0.001. Planned comparisons (with Holm corrections) indicated the interaction was driven by significant differences in accuracy between familiar and novel typefaces, that is, the difference between testing typefaces was larger for the Single-Typeface group (β = 1.44, *SE* = 0.14, *z* = 10.21, 95% CI [1.16, 1.72], *p* < 0.001), than for the Variable-Typeface group (β = 0.65, *SE* = 0.13, *z* = 4.87, 95% CI [0.39, 0.91], *p* < 0.001). However, there was no significant difference in definition accuracy between training groups in either the Familiar (Singe vs Variable: β = 0.46, *SE* = 0.25, *z* = 1.83, 95% CI [− 0.03, 0.96], *p* = 0.136) or Novel Typeface condition (Singe vs Variable: β = − 0.33, *SE* = 0.23, *z* = − 1.46, 95% CI [− 0.77, 0.11], *p* = 0.143). Definition Testing results are depicted in Fig. 7.

### Descriptive results: Form training and testing

**Fig. 8:**
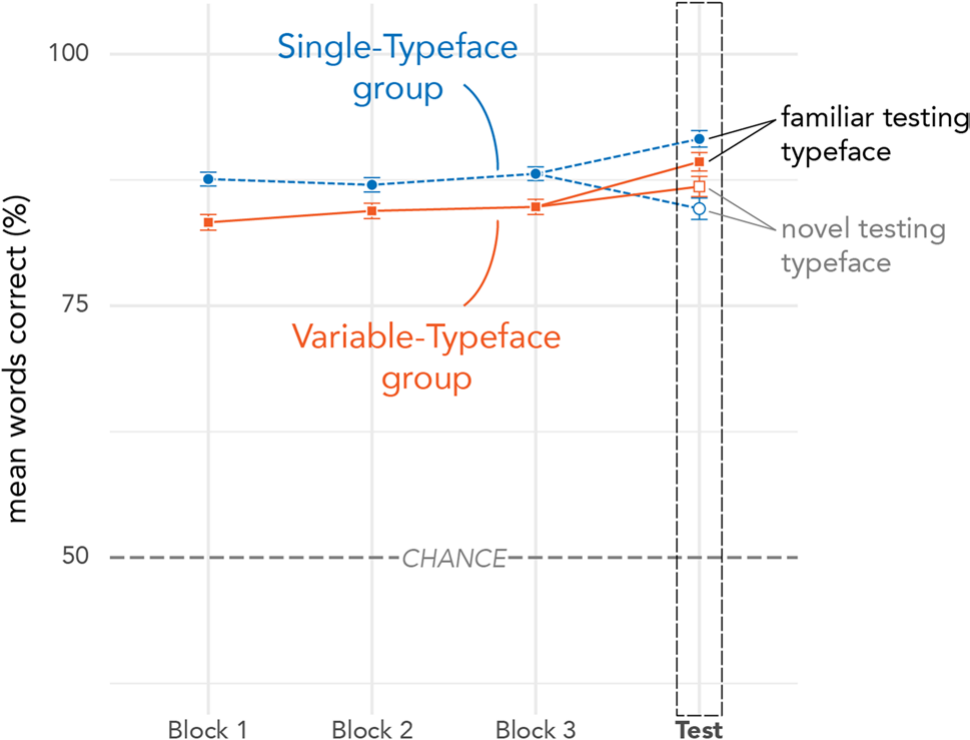
Raw accuracy across blocks of Form Training and Testing. Error bars depict standard error. (For more detailed figures showing individual participant variability, see the Supplementary Materials)

Raw accuracy results across blocks of Form Training are visualized in Fig. 8, testing results are shown in Table 2. As expected, participants in both groups were quite accurate overall. As in Definition Testing, the pattern of results suggests an interaction between training group and testing typeface.

**Table 2:** Form testing accuracy and RT

Training group	Condition	Accuracy		RT	
		Mean (%)	(*SD*)	Mean (ms)	(*SD*)
Single-Typeface	Familiar	91.6	(27.8)	1237	(593)
	Novel	84.6	(36.1)	1813	(962)
	*Overall*	*88.1*	*(32.4)*	*1515*	*(844)*
Variable-Typeface	Familiar	89.3	(30.9)	1367	(690)
	Novel	86.8	(33.8)	1621	(840)
	*Overall*	*88.1*	*(32.4)*	*1492*	*(778)*

**Fig. 9:**
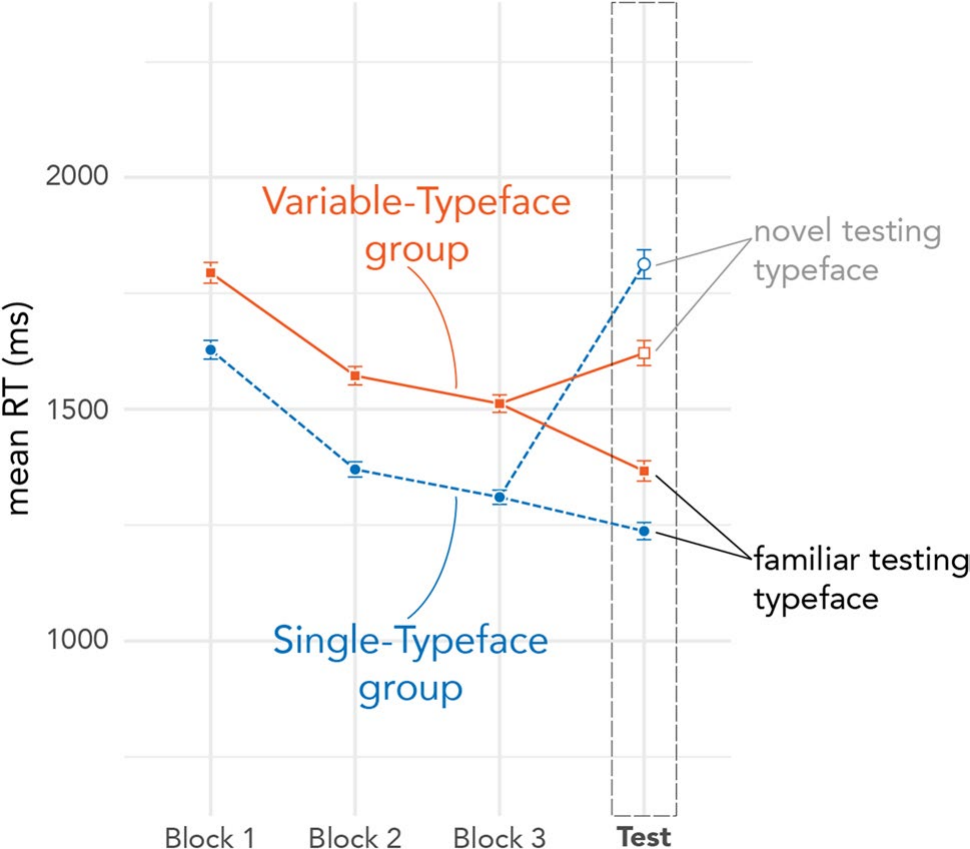
Raw RT across blocks of Form Training and Testing. Error bars depict standard error. (For more detailed figures showing individual participant variability, see the Supplementary Materials)

Figure 9 depicts raw RTs across blocks of Form Training and Testing (also Table 2). Responses from participants in both groups grew faster across blocks. The Single-Typeface group had faster responses across all training blocks compared to the Variable-Typeface group. At Testing, the Single-Typeface group was faster than the Variable-Typeface group for the Familiar Typeface, but slower for the Novel Typeface (even slower than in Block 1 of training). Note that there are no signs of speed-accuracy tradeoffs for either group (higher accuracy pairs with faster RTs).

### Inferential statistical results: Form testing

**Fig. 10:**
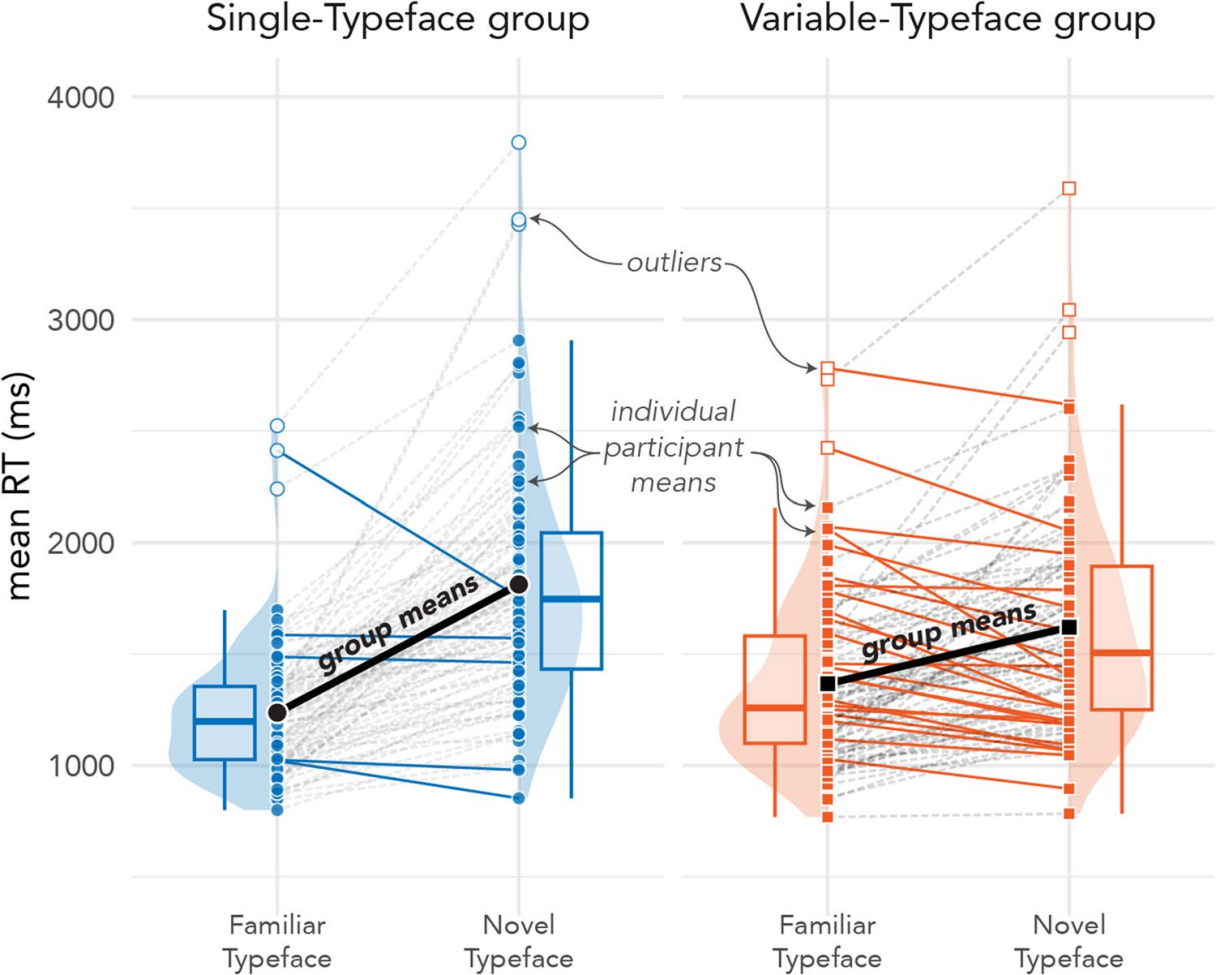
RTs by testing typeface for Form Testing. Single-Typeface group results are pictured on the left; Variable-Typeface on the right. Large black dots indicate group means; smaller dots indicate individual participant means. Lines connect scores for participants/groups in the two testing conditions (Familiar vs Novel Typeface). Colored lines indicate speed-up in RTs; dashed lines indicate slowdown in RTs. The distribution of scores is shown in the shaded area for each testing condition, along with boxplots that capture the median score (thick center line) and interquartile range of scores

Mixed-model ANOVA results for RTs in Form Testing indicated no significant main effect of training group, *F*_(187,57)_ = 0.14, *p* < 0.780, a significant main effect of testing typeface condition, *F*_(37,28)_ = 127.13, *p* < 0.001, and a significant interaction between training group and typeface condition, *F*_(186.42)_ = 44.05, *p* < 0.001. Planned comparisons (with Holm corrections) indicated that the interaction was driven both by significant differences in outcomes for testing typefaces and training groups. All participants were significantly faster when identifying characters tested in the Familiar typeface than in the Novel typeface (Single: β = − 0.36, *SE* = 0.03, *z* = − 13.06, 95% CI [− 0.43, − 0.29], *p* < 0.001; Variable: β = − 0.16, *SE* = 0.03, *z* = − 5.67, 95% CI [− 0.23, − 0.09], *p* < 0.001. Additionally, participants in the Single-Typeface training group were significantly faster than those in the Variable-Typeface group when identifying characters tested in the Familiar typeface (β = − 0.09, *SE* = 0.03, *z* = − 2.83, 95% CI [− 0.17, − 0.01], *p* = 0.006); however, the direction of the effect was reversed for words tested in the Novel typeface, where participants in the Single-Typeface group were significantly *slower* than those in the Variable-Typeface group (β = 0.11, *SE* = 0.04, *z* = 3.02, 95% CI [0.02, 0.21], *p* = 0.006). Form Testing results for individual subjects are depicted in Fig. 10.

## Discussion

This study tested the effects of training with variable typefaces on immediate learning outcomes for Chinese characters. Two groups of participants learned English definitions for 24 Chinese characters, and also learned to differentiate those characters from visually similar lure characters. In Single-Typeface training all characters were presented in the same typeface; in Variable-Typeface training, characters were presented in three different typefaces. For both groups, at testing, half of the words were presented in a familiar typeface and half in a novel typeface. In line with many previous studies on the impacts of variability, results suggest that training with a single typeface produced better immediate outcomes only for that specific typeface, while training with variable typefaces led to better ability to generalize learning to new typefaces that had not previously been encountered.

Definition Testing tested the learning of form-to-meaning associations. Results showed a significant training-by-testing interaction. For Single-Typeface training, participants displayed significantly better knowledge of definitions for characters tested in the familiar typeface relative to the novel typeface. For Variable-Typeface training, the difference between familiar and novel typefaces was not significant. In other words, outcomes were less extreme (in both positive and negative directions) for the Variable-Typeface group than for the Single-Typeface group.

Form Testing tested the speed with which participants could distinguish previously learned characters from visually similar distractors. Results again showed a significant training-by-testing interaction, but this time the effects of variability were clearer. Compared to Single-Typeface training, Variable-Typeface training produced slower responses for characters tested in the familiar typeface, but faster responses in the novel typeface. This provides evidence that training with variable typefaces can result in better generalization of character recognition in newly encountered typefaces.

### Potential explanations for the effects of typeface variability

Along with handwriting and calligraphy, typefaces are a naturally occurring form of visual variability. Previous studies have suggested ways in which typefaces can serve as a useful comparison when testing the influences of handwriting on orthographic learning (e.g., Li & James, 2016; Wiley & Rapp, 2021). The present study suggests that typefaces (or other variable forms of written symbols) may also have utility on their own, particularly as a way to help adult learners of logographic writing systems generalize learning to novel typefaces.

One explanation for this generalization benefit is that training with multiple typefaces provides learners with a wider set of examples to draw from when noticing similarities in unfamiliar typefaces. Although there was no explicit test of similarity in the present study, its importance was tacitly present in the selection of novel typefaces. We intentionally chose two different-looking typefaces (Xing and Yuan) in order to avoid a case where incidental similarity with one training typeface would advantage that training group. An exploratory analysis (recommended by a reviewer and available in Supplemental Materials Appendix F) provides some evidence that similarity may have played a role in transfer to novel testing typefaces. For instance, participants trained only with Hei tested well—even better than the variable group—on the visually similar Yuan typeface (and worse on Xing). What is interesting about the effectiveness of the variable typeface training then, is that overall better generalization occurred despite training providing one-third as many exposures to each training typeface.

Although we framed our study as addressing heterogeneous variability, we acknowledge that the present design confounded *typeface* heterogeneity and set size (‘numerosity’; Raviv et al., 2022). Variable typeface training not only presented more typefaces than single typeface training but it also presented fewer repetitions of each typeface. Importantly, repetitions of the characters themselves were equal in all conditions, and the learning goal for participants was the characters, not the typefaces. Nevertheless, we cannot fully tease apart the effects of typeface repetition and heterogeneity. Future work might examine these issues more directly by testing both heterogeneity and repetition in the same design (cf. Bowman & Zeithamova, 2023).

As noted earlier, recent empirical studies of binary category learning have presented strong rebuffs to the popular impression that high variability training inevitably leads to better generalization than low variability training (Bowman & Zeithamova, 2020, 2023; Hu & Nosofsky, 2024). These studies found that, when total number of training trials was held constant between low and high variability training conditions, low variability training was superior. While the present study also held total trials constant between conditions, it nevertheless does not speak directly to this issue, as it pitted single (‘no variability’) typeface training against variable typeface training. Future work could add additional groups that use more typefaces during training to directly test the high vs low contrast.

At the same time, there are reasons to remain open to the possibility that high typeface variability might be effective for word learning in ways that high variability in the category learning studies was not. The present work differs from those studies in several ways. First, rather than testing the learning of binary categories, this study tested the learning of written words and their meanings (i.e., paired associates). Additionally, those studies often utilized stimuli with rather difficult to identify category features. In contrast, the differences between the 24 Chinese words trained in the present study were generally quite easy to notice in the definition training phase (e.g., 人 vs山 vs 牛). The typefaces that introduced heterogeneous variability were *not* themselves the aim of learning but incidental to the task of learning written symbols. Finally, although participants in the present study had not previously learned Chinese, they were literate adults who implicitly know that typeface variations do not change the identity of written symbols, and could use that knowledge to guide their learning.

Moving away from the effects of variability on its own, another promising outcome in the present study was the benefit of variable typefaces in conjunction with form training, which pushed learners to notice small differences between similar-looking characters. Seyll and colleagues (2020, 2022) have argued that close visual analysis—a necessary part of the process of writing symbols by hand—may play a key role in literacy development. The Form Training approach used in the present study might be one way to encourage close visual analysis for the learning of logographic scripts.

### Practical implications

Put in practical terms, the effects observed in this study were small (accuracy differences of roughly one to two words between training groups; RT differences of several hundred ms), but given that learners of Chinese need to master thousands of characters, these small effects in a lab study might add up to something substantial in the real world. Although one brief lab study is hardly sufficient to suggest broad implications for language teaching, this line of research has potential to make real contributions to Chinese teaching/learning materials by offering a supplement or alternative to the traditional focus on repetitive handwriting for character learning (cf. Chu et al., 2025).

## Limitations

As a first foray into the study of typeface variability, there were naturally many limitations to the present work, some have been mentioned above. Another limitation of the current study is that it only considered learning of form-to-meaning associations in character learning. Future work might consider whether the impacts of visual variability are similar when targeting other aspects of the three-way relationship between the form, sound, and meaning of Chinese characters. One further limitation of the present study is that it targeted only the immediate outcomes of training. Future work can include delayed posttests to determine the durability of training effects.

## Conclusion

This study provides initial evidence that high variability orthographic training can lead to superior generalization of learning—especially form recognition—for novel typefaces. Whereas training with a single typeface produced stronger learning in that one typeface only, training in multiple typefaces appeared to slow down initial learning, but resulted in better generalization of Chinese character learning to previously unencountered typefaces.

## Supplementary Information

Below is the link to the electronic supplementary material.

Supplementary file1 (DOCX 2317 KB)

## Data Availability

All stimuli used in the experiments are included in the paper and its appendices. Data and associated R code for the statistical analyses, as well as supplementary materials are available at: https://osf.io/5b7yn/

## Footnotes

1 Specifically, we used the open source typefaces TW-Kai, Adobe Song Std L, and Noto Sans SC. For the novel typefaces, we used Yuanti SC Light and the open source Ma Shan Zheng Regular.

## Declarations

### Open practices statement

All stimuli used in the experiments are included in the paper and its appendices. Data and associated R code for the statistical analyses, as well as supplementary materials are available at: https://osf.io/5b7yn/
