# Supplementary material for: High variability orthographic training: Learning words in a logographic script through training with multiple typefaces
Source: Psychon Bull Rev. 2025 Mar 17;32(5):2090–103. doi: 10.3758/s13423-025-02646-0 (PMC12426071; doi:10.3758/s13423-025-02646-0)
Supplement: Supplementary file 1 — Supplementary file1 (DOCX 2317 KB) [file 13423_2025_2646_MOESM1_ESM.docx]

**Appendix A: All stimuli (targets and lures in all typefaces)**


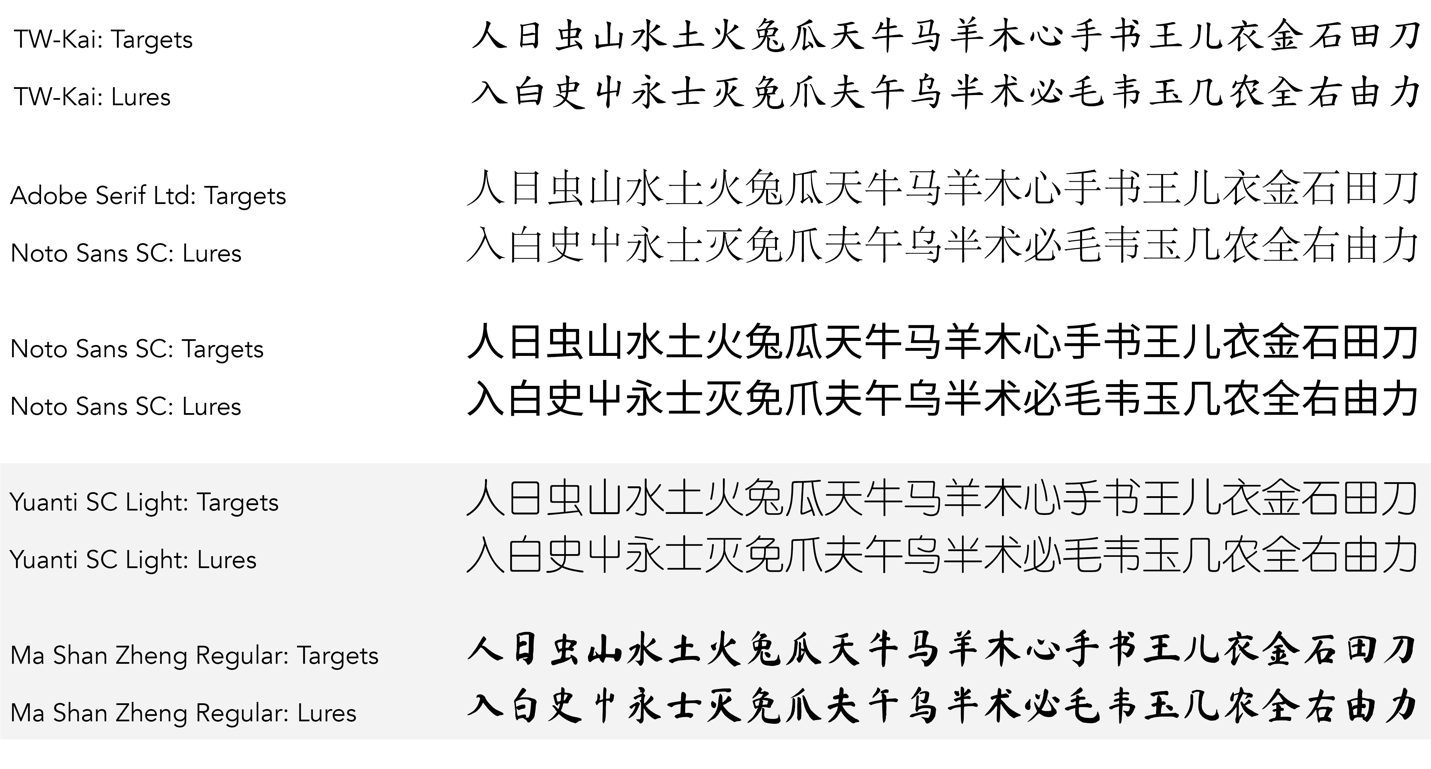


**Appendix B: Additional details on participants, sub-groups, and experimental lists**

*Participant language background*

Participation was not restricted on the basis of first or second language experience except that participants had to indicate they have never learned a *written* language using a non-roman alphabet. The critical question (on the post-study questionnaire) used for exclusion was: *“Have you ever studied a language with a non-roman alphabet? Examples include Hebrew, Russian, Sanskrit, Chinese, Korean, and so on.”* 187 of the 190 participants responded ‘no’. Three others responded ‘yes’, but upon checking their responses, it was determined they should have responded ‘no’: in a follow-up question about the non-roman alphabet they had learned, one person listed French, one Italian, and one Spanish.

Among the 190 participants who were included in the study, all but one reported English as their first language. That individual indicated Vietnamese as a first language. This person was born and grew up in the United States, and presumably learned Vietnamese as a heritage/home language (note that modern Vietnamese does use a roman alphabet). Thirty-one participants reported learning another language during childhood. The fourteen other languages included Cantonese, French, German, Haitian Creole, Indonesian, Italian, Krio, Polish, Spanish, Sign Language, Telugu, Vietnamese, Yoruba, and ‘an isolated Chinese dialect’. Cantonese and a ‘Chinese dialect’ could, indeed, be written with Chinese characters, and Telugu uses a non-roman alphabetic script. Yoruba and Indonesian are also sometimes written using non-roman alphabetic scripts. However, in all of these cases, participants responded negatively to the critical question about non-roman scripts. Inspection of individual performance for these participants also showed no obvious knowledge of Chinese (i.e., there was no immediate recognition of words during training). All of the relevant participants indicated that they were born and grew up in the United States. Based on answers to other questions about language background, it seems these languages were all heritage/home languages, perhaps learned only as spoken languages. Sixty-eight additional participants indicated having learned ASL, French, German, Hindi, Italian, Latin, or Spanish as a second language.

*Sub-groups and lists within each training type*

Participants were distributed as evenly as possible across training sub-groups. For Variable Typeface training, three basic lists were used that differed in which typeface (Hei, Kai, Song) appeared first and last among Definition Training blocks, and first in Form Training blocks.

*Number of participants per sub-group*

| Single | Hei | 32 |
| --- | --- | --- |
| Single | Kai | 32 |
| Single | Song | 31 |
| Variable | (Hei first) | 32 |
| Variable | (Kai first) | 31 |
| Variable | (Song first) | 32 |

Additionally, half of the participants in each subgroup had the novel Yuan typeface for Definition Testing and Xing for Form Testing, and half Xing for Definition Testing and Yuan for Form Testing.

Finally, the specific vocabulary items that appeared in the familiar/novel typeface were also counter-balanced across lists.

In total, there were 24 unique lists, twelve for type of training (Single vs Variable). The order of characters within blocks of training and testing was randomized.

**Appendix C: Additional Figures**

Below I provide additional figures that show the variability and distributions of participants performance in the two training and testing phases.

**DEFINITION TRAINING AND TESTING ACCURACY (Figure B1)**


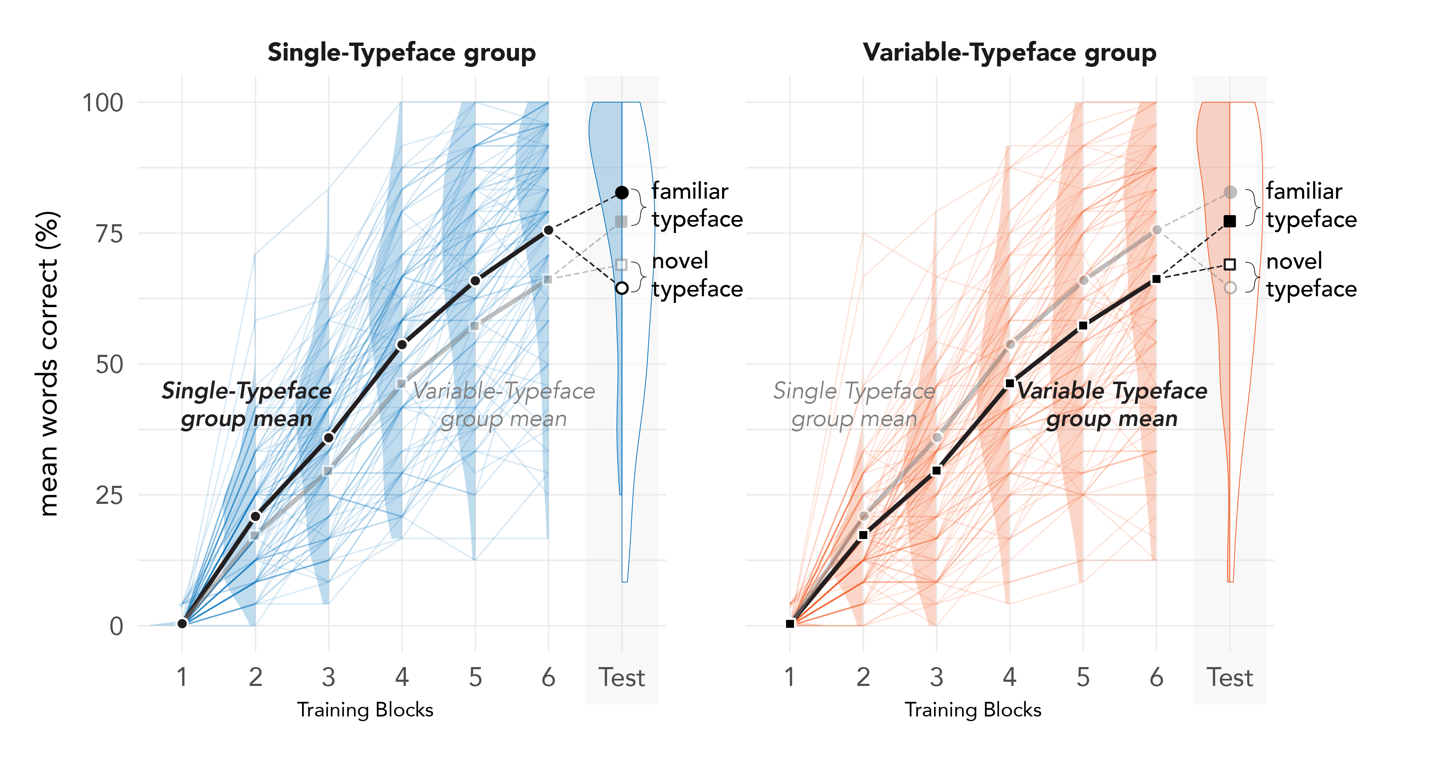


**Figure B1.** Raw accuracy across blocks of Definition Training and Testing. Single Typeface results are pictured on the left, Variable Typeface on the right, with the average of the other group presented in grey as a reference. Thin colored lines indicate individual participant scores, with the distribution of scores shown in the shaded area for each block. The distribution of results in the Test are contrasted by color (Familiar Typeface) and white (Novel Typeface).

**DEFINITION TESTING ACCURACY (Figure B2)**


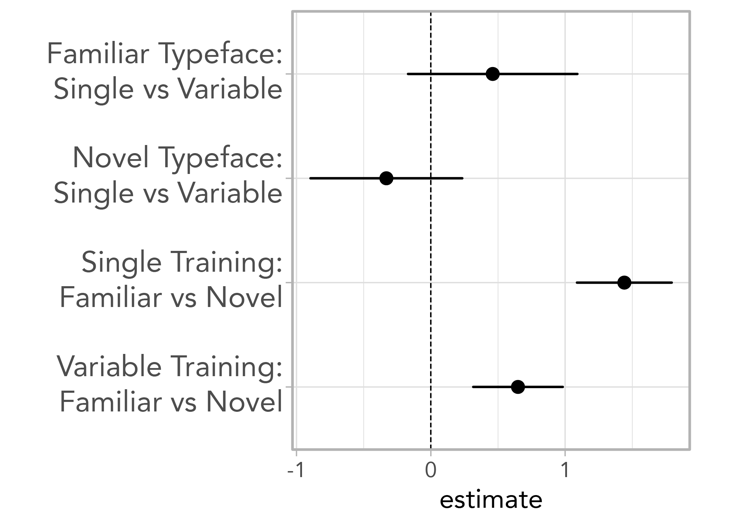


**Figure B2.** Asymptotic 95% Confidence Intervals (with Holm corrections) for planned comparisons of accuracy in Definition Testing.

**FORM TRAINING AND TESTING ACCURACY (Figure B3)**


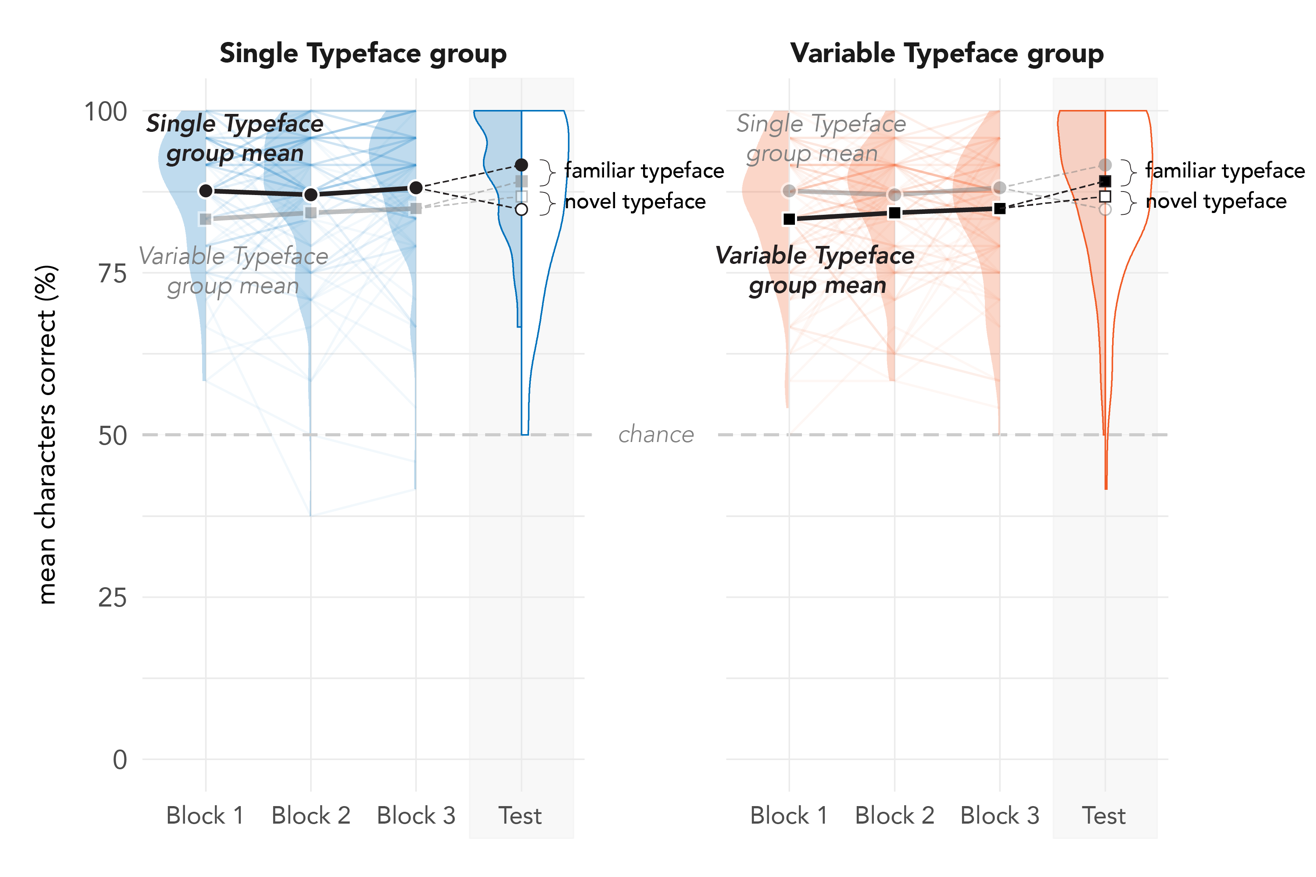


**Figure B3.** Raw accuracy across blocks of Form Training and Testing. Single Typeface results are pictured on the left, Variable Typeface on the right, with the average of the other group presented in grey as a reference. Thin colored lines indicate individual participant scores, with the distribution of scores shown in the shaded area for each block. The distribution of results in the Test are contrasted by color (Familiar Typeface) and white (Novel Typeface).

**FORM TRAINING AND TESTING RTs (Figure B4)**


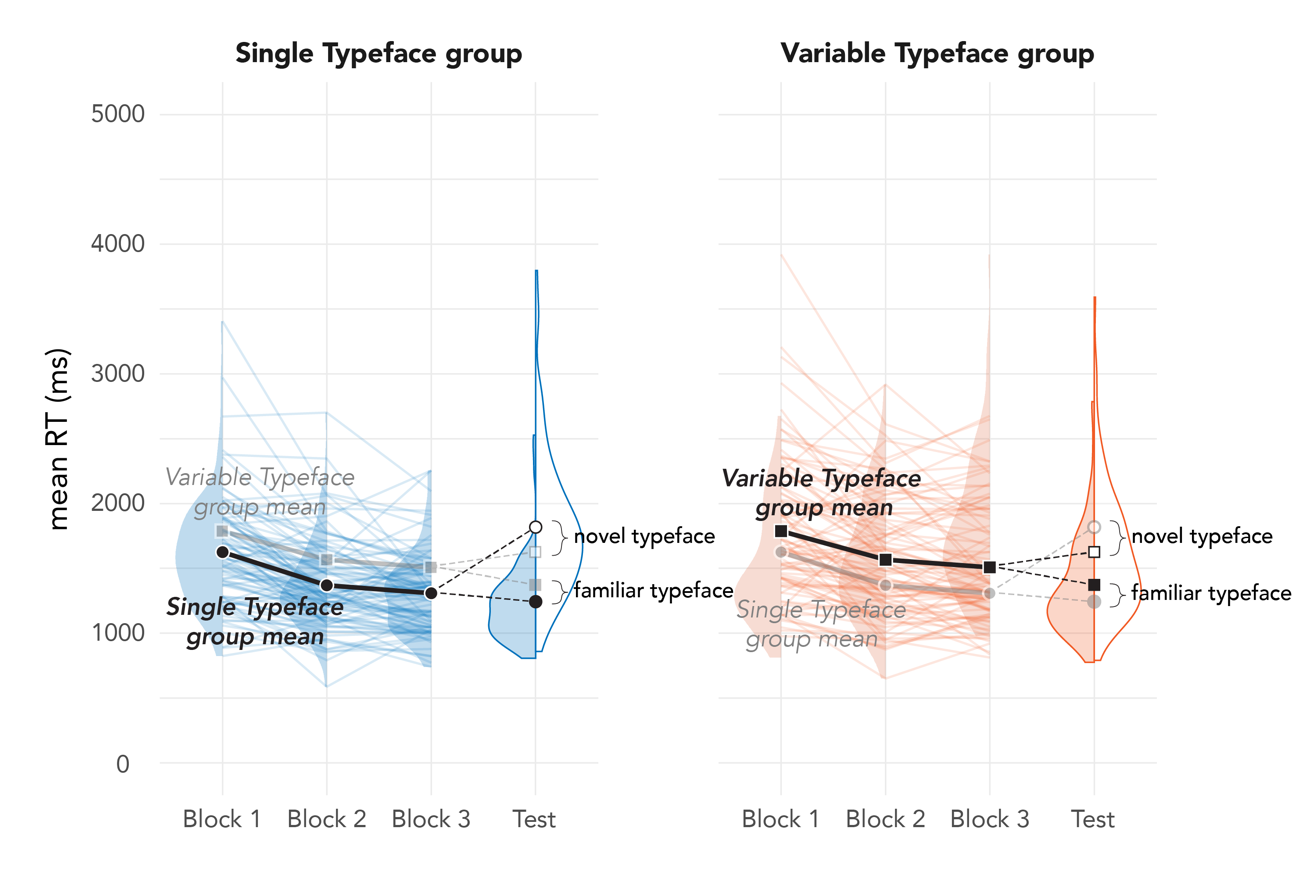


**Figure B4.** Raw RTs across blocks of Form Training and Testing. Single Typeface results are pictured on the left, Variable Typeface on the right, with the average of the other group presented in grey as a reference. Thin colored lines indicate individual participant scores, with the distribution of scores shown in the shaded area for each block. The distribution of results in the Test are contrasted by color (Familiar Typeface) and white (Novel Typeface).

**FORM TESTING RTs (Figure B5)**


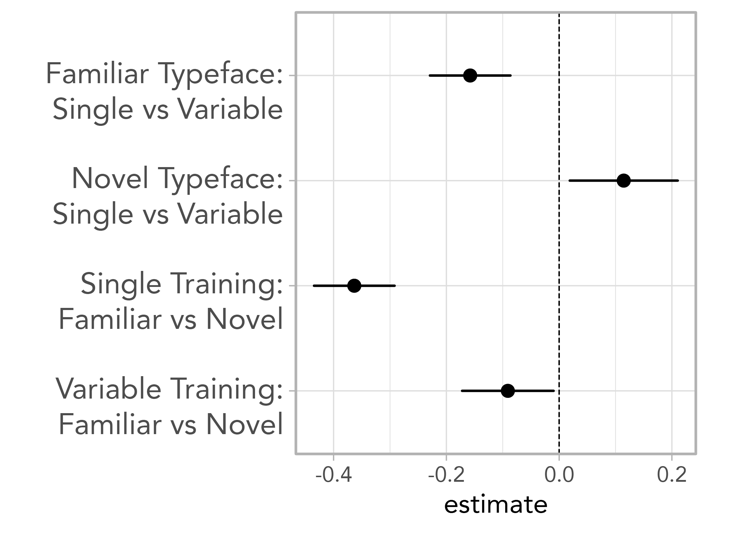


**Figure B5.** 95% Confidence Intervals (with Holm corrections) for planned comparisons of RTs in Form Testing.

**Appendix D: Analysis of Accuracy Results from Form Testing**

All statistical analyses were conducted in *R* (version 4.0.3, R Core Team, 2022). Accuracy data for Form Testing was modeled using generalized linear mixed-effects regression. All models were fit with the *lme4* (version 1.1.21, using the BOBYQA optimizer, Bates et al., 2015), and *afex* (Singmann et al., 2022) packages. The dependent variable was Accuracy (1,0). Fixed effects were sum coded (1, -1) and included the factors Training Group (Single, Variable), and Typeface Condition (Familiar, Novel). Random intercepts and slopes were included for the effects of participants and items. The fully-specified maximal random effects model was fit first (Barr et al., 2013; Bates et al., 2015) using the *mixed()* function from *afex*. If a model failed to converge or generated singular fit warnings, it was refit using a zero-correlation parameter, and the random components that generated the smallest variances were dropped. The maximal model that converged with no singular fit warnings was retained as the final model.

The final model for Form Testing accuracy included by-subject intercepts and slopes for the effect of typeface condition, and by-item intercepts: *mixed/glmer model formula*: accuracy ~ 1 + training group * testing typeface + (1 + typeface condition | participant) + (1 | item).

Mixed Model ANOVA results for accuracy in Form Testing indicated no significant main effect of training group (χ^2^_(1)_=0.02, *p*<.902), a significant main effect of testing typeface (χ^2^_(1)_=4.22, *p*=.040), and a significant interaction between training group and testing typeface (χ^2^_(1)_=6.17, *p*<.013). It is noted that the interaction was not significant in the fully specified model (which generated singular fit warning), and so should be treated with additional skepticism. Planned comparisons (with Holm corrections) suggested that the interaction was driven by a significant difference in accuracy between familiar and novel test typefaces for the Single Typeface group. That is, the Single Typeface group was significantly more accurate when identifying characters in a familiar typeface than in a novel typeface (familiar vs novel typeface: 𝛽=0.66, *SE*= 0.20, *z*=3.24, *95% CI*=[0.14, 1.19], *p*=.005); for the Variable Typeface group, there was no significant difference between testing typefaces (familiar vs novel typeface: 𝛽=0.14, *SE*=0.20, *z*=0.69, *95% CI*=[-0.38, 0.66], *p*=.490). Furthermore, there was no significant difference in form recognition accuracy between training groups in either the Familiar (Single vs Variable: 𝛽=0.28, *SE*= 0.19, *z*=1.50, *95% CI*=[-0.20, 0.76], *p*=.399) or the Novel typeface condition (Single vs Variable: 𝛽=-0.24, *SE*=0.18, *z*=-1.31, *95% CI*=[-0.71, 0.23], *p*=.399). 95% A visualization of accuracy results is shown in Figure C1. Confidence Intervals for the interaction and post-hoc comparisons are displayed in Figure C2.


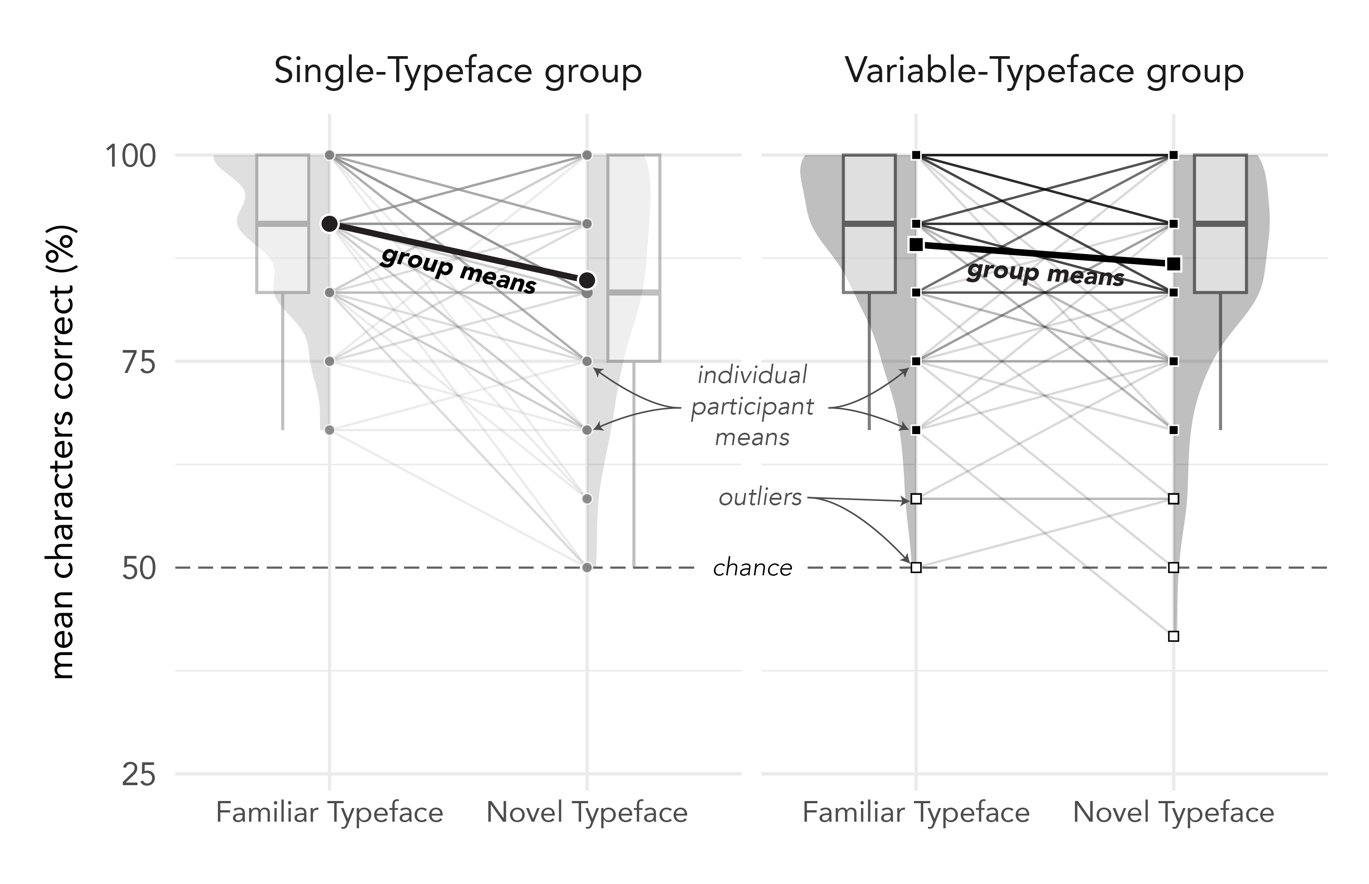


**Figure C1.** Accuracy by testing typeface for Form Testing. Single-Typeface group results are pictured on the left, Variable-Typeface on the right. Large black dots indicate group means, smaller dots indicate individual participant means. Lines connect scores for participants/groups in the two testing conditions (Familiar vs Novel Typeface). The distribution of scores is shown in the shaded area for each testing condition, along with boxplots that capture the median score (thick center line) and interquartile range of scores.


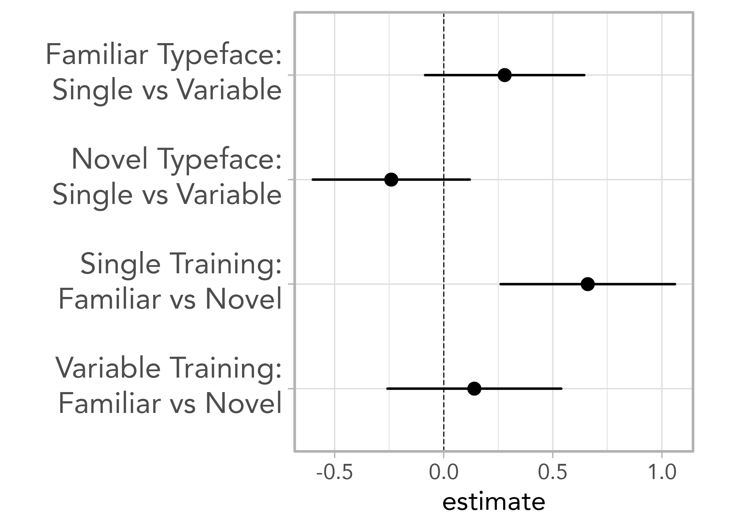


**Figure C2.** 95% Confidence Intervals (with Holm corrections) for planned comparisons of accuracy in Form Testing.

**Appendix E: Alternative Analysis of Accuracy Results from Definition Testing (counting apparent typos as correct answers)**

In order to test whether the automatic scoring of definitions had an impact on outcomes, the Definition Test data was recoded so that the 47 apparent typos (manually identified) were counted as correct. This slightly raised the mean scores across the tests (Table D1)

**Table D1.** Comparison of mean accuracy for Definition Testing before and after counting apparent typos as ‘correct’ answers.

|  |  | Reported in manuscript | | **With typos scored ‘correct’** | |
| --- | --- | --- | --- | --- | --- |
| Training Group | Condition | Mean (%) | (SD) | **Mean (%)** |  |
| Single-Typeface | Familiar | 82.6 | (37.9) | **83.7** |  |
| *(n=95)* | Novel | 64.3 | (47.9) | **65.3** |  |
|  | *Overall* | *73.5* | *(44.2)* | ***74.5*** |  |
| Variable-Typeface | Familiar | 77.4 | (41.9) | **78.4** |  |
| *(n=95)* | Novel | 69.1 | (46.2) | **70.2** |  |
|  | *Overall* | *73.2* | *(44.3)* | ***74.3*** |  |

Using this updated data, alternative models were run following the procedures outlined in the main manuscript. Results were substantively the same as in the original analysis. There was a statistically significant effect of testing typeface, and a significant interaction between testing typeface and training group.

Mixed Model Anova Table (Type 3 tests, LRT-method)

Model: ACC ~ training_type * font_condition + (1 + font_condition |

Model: participantID) + (1 | definition)

Data: defs

Df full model: 8

Df Chisq Chi Df Pr(>Chisq)

Training group 7 0.0377 1 0.846

Test typefc 7 88.3987 1 < 2.2e-16 ***

Training group:test typefc_ 7 18.2343 1 1.953e-05 ***

Planned comparisons (with Holm corrections) indicated the interaction was driven by significant differences in accuracy between familiar and novel typefaces.

Estimate Std.Err z Pr(>|z|)

Var:Fam-Novel 0.7034 0.1406 5.001 1.71e-06 ***

Sing: Fam-Novel 1.5445 0.1482 10.419 < 2e-16 ***

Novel:SvV -0.3752 0.2376 -1.579 0.166

Familiar:SvV 0.4659 0.2689 1.733 0.166

**Appendix F: Exploratory analysis: Effects of training-to-testing typeface similarity**

This analysis scrutinizes the outcomes of training a bit more closely to interrogate the role of similarity/difference between training and testing typefaces (this analysis was suggested by a reviewer). Specifically, if we believe that similarity between typefaces might play some role in the generalization from training to testing, the available data allow us to explore whether that is indeed the case. As this analysis was not planned, and would be statistically underpowered, no inferential statistical tests are applied below. Instead, we simply consider suggestive patterns in the descriptive statistics which might suggest directions for future targeted investigation.

The intuition explored here is that similarities between training and testing typefaces are likely to play a substantial role in whether or not participants can transfer training to testing. For instance—based on subjective impressions of Figure E1 below—the Yuan testing typeface appears more similar to the Hei typeface than it does to the Kai typeface; the Xing testing typeface appears more similar to Kai than it does to Hei. This suggests that the subgroup of Single-Typeface training participants that were trained with Hei will be more accurate when tested with Yuan than the sub-group trained with Kai (and vice-versa for testing with Xing). It may even be the case that this specificity of transfer would lead to *better performance for those subgroups than for the Variable-Typeface group*. That is, the Hei-trained subgroup would outperform the Variable-Typeface group when tested with Yuan, and the Kai-trained subgroup would outperform the Variable-Typeface group when tested with Xing.


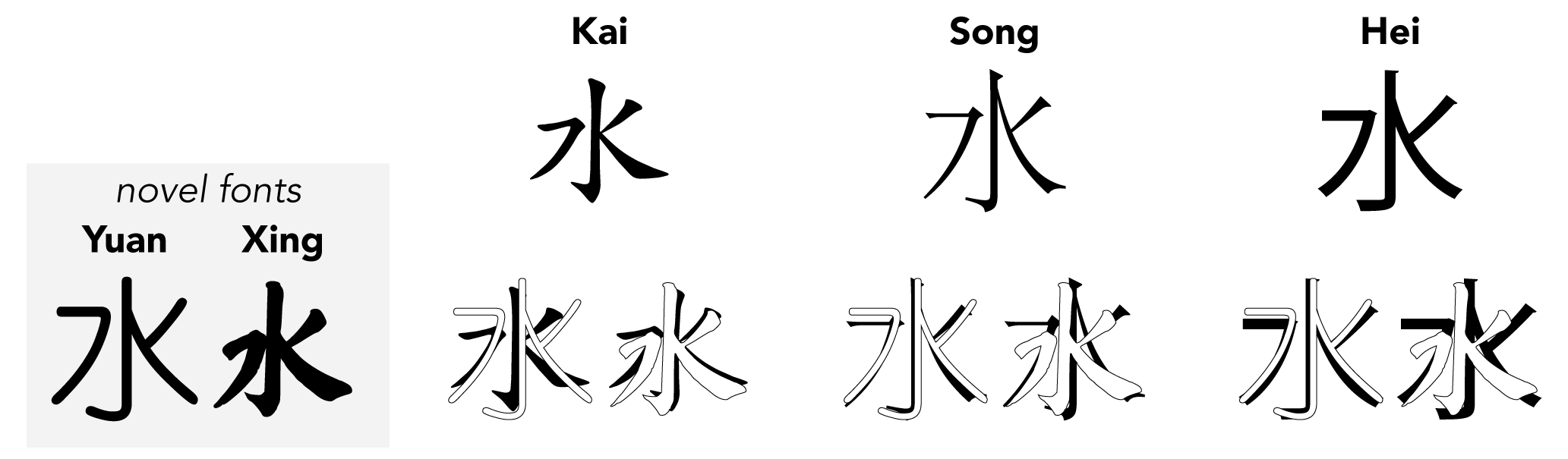


Figure E1. Training typefaces overlaid with novel testing typefaces for ‘water’

Indeed, these predictions appear to be borne out by the descriptive patterns for mean accuracy in both Definition (Table E1) and Form testing (Table E2). However, the same is not true for the RT data (Table E2, right columns), which shows the Song-trained and Variable-Typeface groups having faster RTs than the Hei group; and the Variable-Typeface group having similar or slightly faster RTs compared to the Kai-trained group. All of these observations should be strongly tempered by recognition that the data at this level are quite sparse, and individual participant performance could easily skew the averages (the sparseness can be seen in Figure E2-E4 below).

**Table E1:** Definition Testing accuracy by sub-group (training typeface)

| Group | Trained | Tested | Mean (%) | (SD) |
| --- | --- | --- | --- | --- |
| single | Hei | Yuan | 79.7 | (40.3) |
| single | Kai | Yuan | 67.7 | (46.9) |
| single | Song | Yuan | 71.9 | (45.1) |
| variable | *all* | Yuan | 74.0 | (43.8) |
|  |  |  |  |  |
| single | Hei | MSZ | 49.5 | (50.1) |
| single | Kai | MSZ | 66.7 | (47.3) |
| single | Song | MSZ | 49.4 | (50.1) |
| variable | *all* | MSZ | 64.2 | (48.0) |


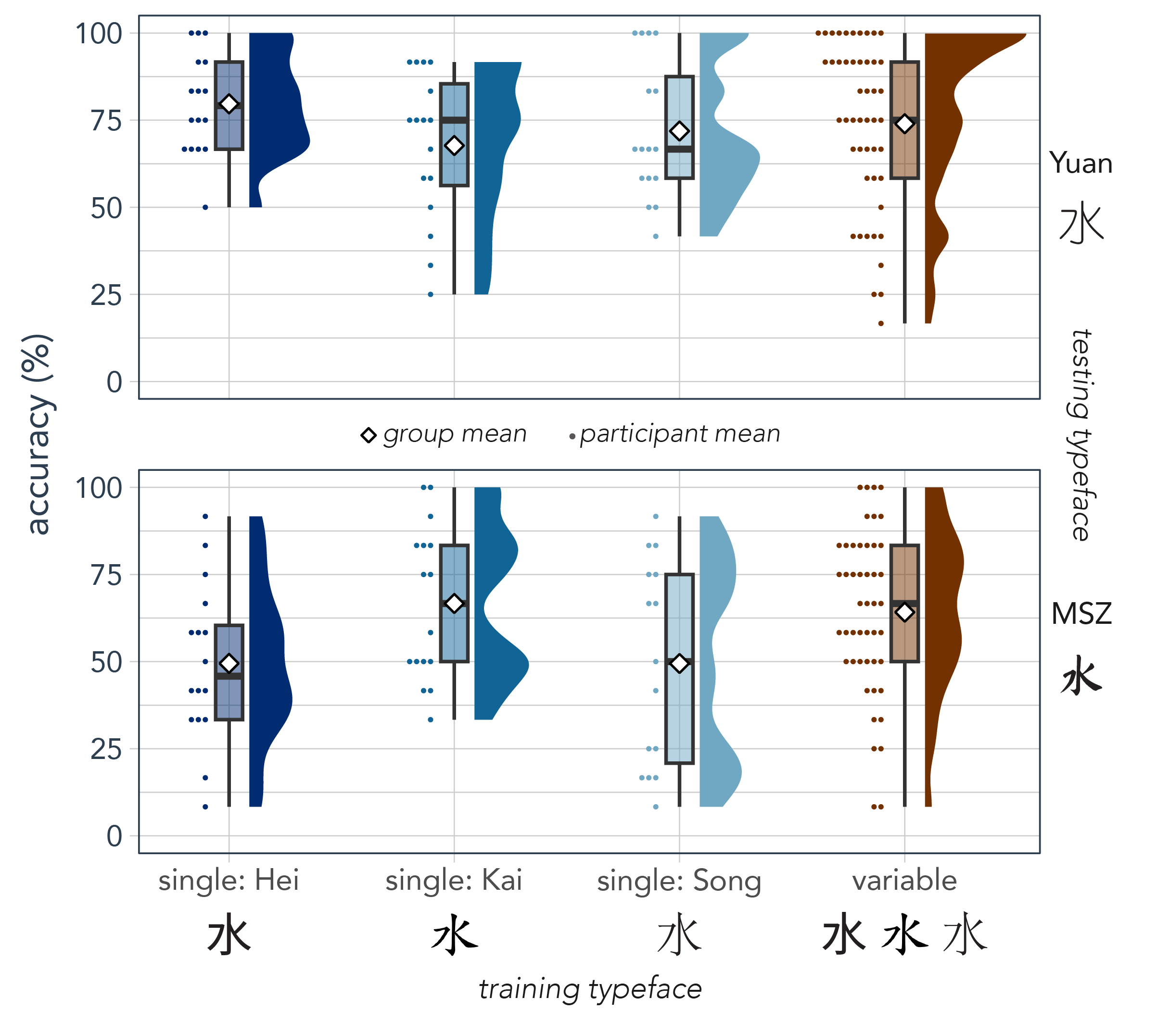


**Figure E2.** Accuracy results of Definition Testing according to training and testing typeface

**Table E2:** Form Testing accuracy and RT by sub-group (training typeface)

|  |  |  | Accuracy | |  | RT | |
| --- | --- | --- | --- | --- | --- | --- | --- |
| Group | Trained | Tested | Mean (%) | (SD) |  | Mean (ms) | (SD) |
| single | Hei | Yuan | 89.1 | (31.3) |  | 1667 | (880) |
| single | Kai | Yuan | 80.7 | (39.5) |  | 2035 | (950) |
| single | Song | Yuan | 83.3 | (37.4) |  | 1587 | (746) |
| variable | *all* | Yuan | 86.6 | (33.9) |  | 1590 | (806) |
|  |  |  |  |  |  |  |  |
| single | Hei | MSZ | 82.8 | (37.8) |  | 1889 | (1164) |
| single | Kai | MSZ | 89.6 | (30.6) |  | 1686 | (877) |
| single | Song | MSZ | 82.3 | (38.3) |  | 2022 | (1019) |
| variable | *all* | MSZ | 87.0 | (33.6) |  | 1652 | (849) |


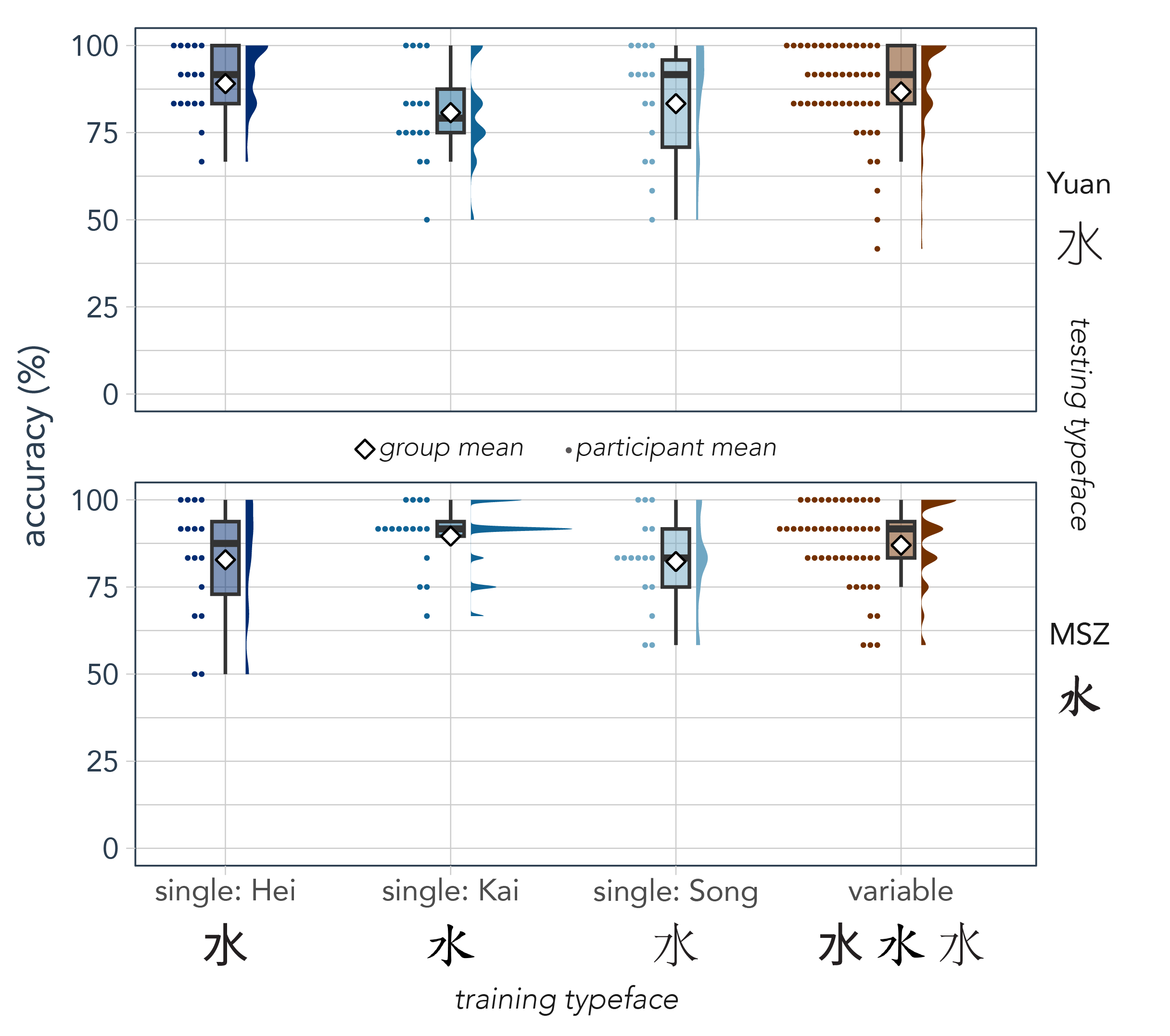


**Figure E3.** Accuracy results of Form Testing according to training and testing typefaces


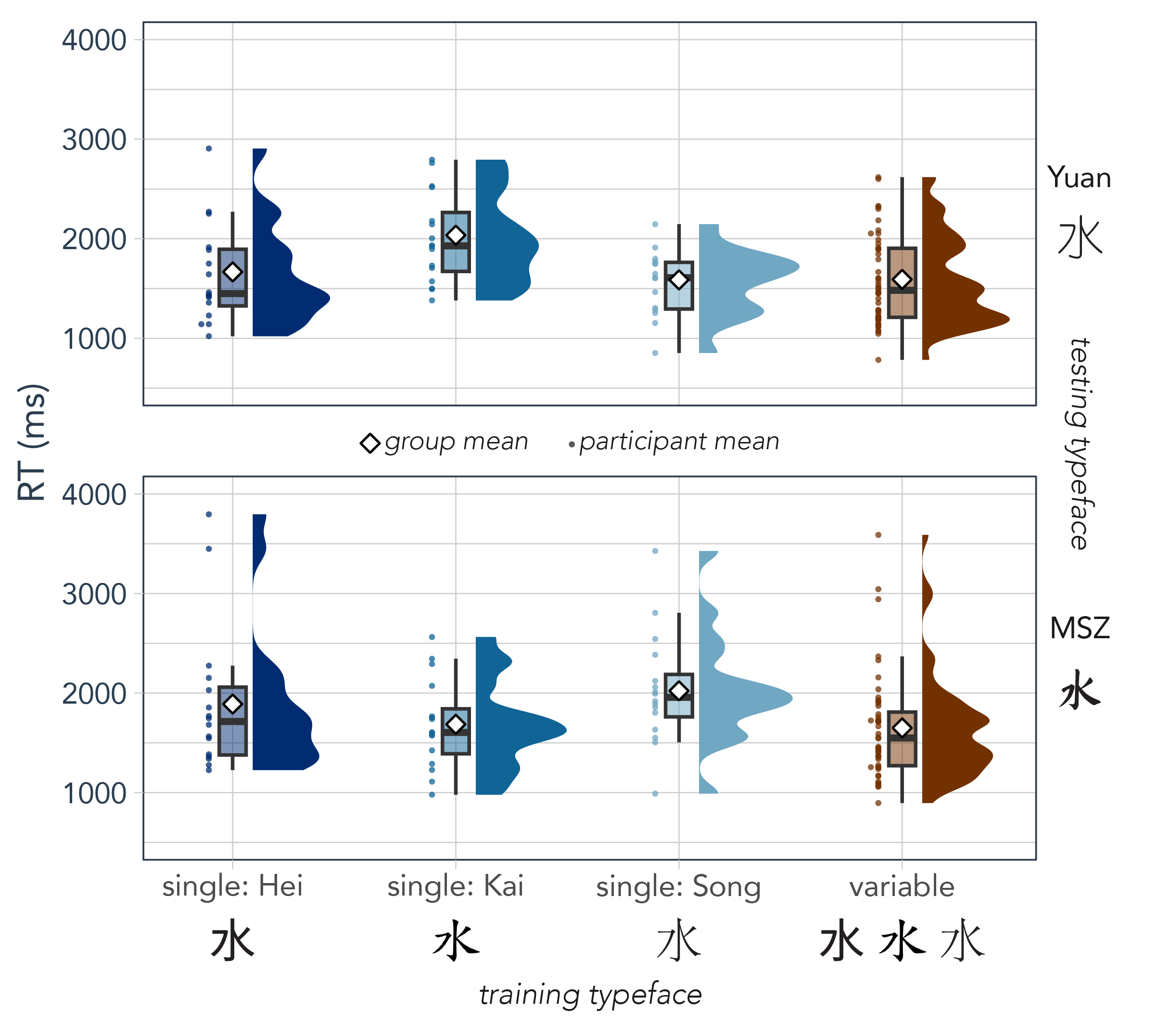


**Figure E4.** RT results of Form Testing according to training and testing typefaces
